# Defensive Mechanisms in Cucurbits against Melon Fly (*Bactrocera cucurbitae*) Infestation through Excessive Production of Defensive Enzymes and Antioxidants

**DOI:** 10.3390/molecules26216345

**Published:** 2021-10-20

**Authors:** Madhusudana Somegowda, S. Raghavendra, Shankarappa Sridhara, Achur. N. Rajeshwara, Siddanakoppalu. N. Pramod, S. Shivashankar, Feng Lin, Tarek K Zin El-Abedin, Shabir Hussain Wani, Hosam O. Elansary

**Affiliations:** 1 Department of Biochemistry, University of Agricultural and Horticultural Science, Shivamogga 577204, Karnataka, India; ysmadhu84@gmail.com (M.S.); raghu.rsn2004@gmail.com (S.R.); 2 Department of Studies and Research in Biochemistry, Jnana Shayadri, Kuvempu University, Shimoga 577203, Karnataka, India; rajachur@gmail.com; 3 Center for Climate Resilient Agriculture, University of Agricultural and Horticultural Science, Shivamogga 577204, Karnataka, India; sridharas1968@gmail.com; 4 Department of Studies in Biochemistry and Food Technology, Davanagere University, Shivagangotri, Davanagere 577007, Karnataka, India; 5 Department of Plant Physiology and Biochemistry, IIHR, Hesaraghatta, Bangalore 560089, Karnataka, India; siva@iihr.ernet.in; 6 Department of Plant, Soil and Microbial Sciences, Michigan State University, East Lansing, MI 48824, USA; fenglin@msu.edu; 7 Department of Agriculture & Biosystems Engineering, Faculty of Agriculture (El-Shatby), Alexandria University, Alexandria 21545, Egypt; drtkz60@gmail.com; 8 Mountain Research Centre for Field Crops, Sher-e-Kashmir University of Agricultural Sciences and Technology of Kashmir, Shalimar 181101, Srinagar, India; shabirhussainwani@gmail.com; 9 Plant Production Department, College of Food & Agriculture Sciences, King Saud University, Riyadh 11451, Saudi Arabia

**Keywords:** melon fly, ROS, antioxidant, defensive enzymes, phenols and flavonoids, superoxide dismutase

## Abstract

Melon fly (*Bactrocera cucurbitae*) is the most common pest of cucurbits, and it directly causes damage to cucurbit fruits in the early developmental stage. The infection of fruit tissues induces oxidative damage through increased generation of cellular reactive oxygen species. The effects of melon fly infestation on the production of defensive enzymes and antioxidant capabilities in five cucurbit species, namely, bottle gourd, chayote, cucumber, snake gourd, and bitter gourd, were investigated in this study. The total phenolic and flavonoid content was considerably higher in melon fly infestation tissues compared to healthy and apparently healthy tissues. The chayote and bottle gourd tissues expressed almost 1.5- to 2-fold higher phenolic and flavonoid contents compared to the tissues of bitter gourd, snake gourd, and cucumber upon infestation. Defensive enzymes, such as peroxidase (POD), superoxide dismutase (SOD), polyphenol oxidase (PPO), and catalase (CAT), were high in healthy and infected tissues of chayote and bottle gourd compared to bitter gourd, snake gourd, and cucumber. The activity of POD (60–80%), SOD (30–35%), PPO (70–75%), and CAT (40–50%) were high in infected chayote and bottle gourd tissue, representing resistance against infestation, while bitter gourd, snake gourd, and cucumber exhibited comparatively lower activity suggesting susceptibility to melon fly infection. The antioxidant properties were also high in the resistant cucurbits compared to the susceptible cucurbits. The current research has enlightened the importance of redox-regulatory pathways involving ROS neutralization through infection-induced antioxidative enzymes in host cucurbit resistance. The melon fly infestation depicts the possible induction of pathways that upregulate the production of defensive enzymes and antioxidants as a defensive strategy against melon fly infestation in resistant cucurbits.

## 1. Introduction

Cucurbits are members of the Cucurbitaceae family, which are mostly consumed as food around the world. A variety of cucurbit cultivars are grown in India, which accounting for approximately 5.6% of the total fruit’s production. Every year, approximately 30–40% of the fruits are lost due to the fact of various pathogens and insects that damage the vegetative growth and development of the fruits [1]. The melon fly (cucurbit fruit fly), which is called the *Bactrocera cucurbitae* (Coquillett) (Diptera: Tephritidae), consists of 4000 species with a wide distribution in tropical, subtropical, and temperate regions across the globe [2]. It is a serious pest of cucurbitaceous fruits, causing damage to 81 host plants with the extent of fruit loss varying between 30% and 100% depending on the species and season of occurrence [3]. It prefers young, green, soft-skinned fruits for infestation. The eggs are inserted 2–4 mm deep into the fruit tissues, and the maggots feed inside the fruit, causing harm to the infected fruits and economic loss [3]. Some species of cucurbit fruits, such as chayote and bottle gourd, exhibit resistance to melon fly infestation with very minor damage, whereas most others are highly susceptible and can undergo damage resulting in 90% fruit loss in a crop season. The susceptibility and resistibility to melon fly infestation might be accredited to biochemical constituents expressed in response to infection [4]. Many studies have reported on the defensive and repair mechanisms based on biochemical events occurring in fruit tissues due to the presence of melon fly infestation [5,6].

One reason for the development of secondary compounds in plants and fruits is the result of their co-existence with insects. They constitute an essential defense arsenal component in plants that otherwise lack an immune system [7]. They are ubiquitous in all plant parts and have been implicated in imparting resistance to plants against insect pests [8]. The cucurbits fruits contain various phenolic compounds produced as secondary metabolites and are reported to have antioxidant potential [9]. These also defend and provide immunity from external infectious agents to some extent [10]. They protect the plant tissues from damage induced by reactive oxygen species generated during the cellular metabolism [11]. Cucurbitaceae fruits present various phytochemicals such as tannins, glycosides, terpenoids, resins, saponins, carbohydrates, carotenoids, and phytosterols [12]. The melon fly infection in cucurbit fruit induces damage to fruit tissues, resulting in tissue inflammation and wounds [13]. This generates reactive oxygen species (ROS) that causes an oxidative burst of cells and increases tissue damage [14]. In response to the inflammatory response during the infection, the tissue synthesizes phenolics and flavonoids, which non-enzymatically scavenges and reduces oxidative damage. The cells also increase the number of antioxidant enzymes [15], which helps to reduce inflammation, scavenge cellular reactive molecules, and also in tissue remodeling and repair [16,17]. The present study aimed at studying these enzymatic and non-enzymatic antioxidants.

The potential of the selected resistant fruits—chayote (*Sechium edule*) and bottle gourd (*Lagenaria siceraria*)—and the susceptible cucurbits fruits —cucumber (Cucumis sativus), bitter gourd (*Momordica charantia*), and snake gourd (*Trichosanthes cucumerina*)—in response to melon fly infestation was studied. The study demonstrated the potential of antioxidants in damaged cells to resist the infection to protect the fruits.

## 2. Results

Melon fly eggs were ovipositioned on healthy tissues of Cucurbitaceae fruits and monitored for hatching and larval feeding to induce damage. Based on the damage, the infection was graded as healthy, apparently healthy, and infected, and they were used for biochemical studies to understand susceptibility and resistance against melon fly infestation. The healthy and infected tissues were selected for examination and graded depending on the overpictured and tissue degeneration. Susceptible fruits appeared to have large damaged portions compared to resistant fruits. 

### 2.1. Accelerated ROS Detoxifying Potential as A Defensive Response against Melon Fly Infection in Cucumber and Chayote

Melon fly infestation resulted in pronounced phenolic and flavonoid contents in cucurbit fruits. A significant increase in phenolic contents was observed in chayote fruit tissue than in cucumber tissue upon melon fly infestation. Compared to APH (apparently healthy) tissue and HT (healthy tissue), chayote infected tissue had a high phenolic content. On the other hand, cucumber showed high phenolic content in INF (infested) tissue compared to APH and HT. When chayote fruit tissue was infected, the flavonoids were substantially higher than in cucumber tissue. Chayote synthesized more flavonoids as secondary metabolites in response to melon fly infestation (Table 1). These are synthesized as a secondary metabolite in response to melon fly infection, which protects against oxidative damage to the tissue. Total phenolic and flavonoid contents were greater in bottle gourd than in bitter gourd and snake gourd, potentially indicating bottle gourd fruits’ resistance ability (Table 1). The phenolic and flavonoid contents were higher in infected bottle gourd than healthy samples, and flavonoid contents were higher in infected bottle gourd than in healthy and apparently healthy samples, whereas its contents decreased in bitter and snake gourds.

### 2.2. Non-Enzymatic Antioxidant Potential of Healthy and Infected Cucurbits

#### 2.2.1. Superoxide Anion (o2–) Assay

**Figure 1:**
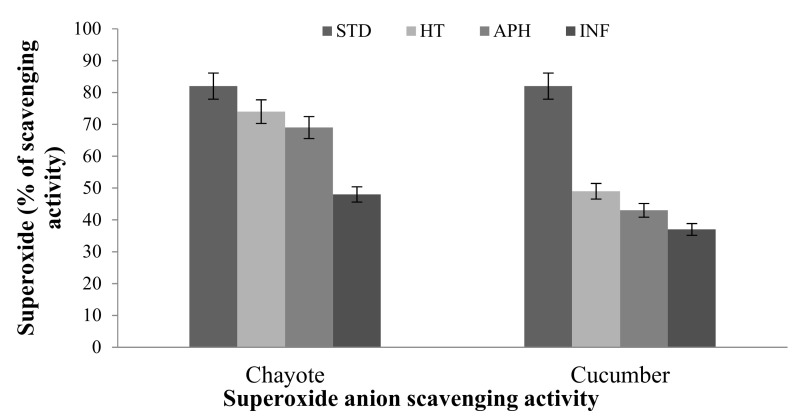
The superoxide anion antioxidant activity of melon fly infection in healthy (HT), apparently healthy (APH), and infected (INF) tissue extracts of cucurbit fruit. The vertical bars indicate the standard error. Significance at *p* ≤ 0.05.

Superoxide anion activity showed statistically significant results with the melon fly infestation. Superoxide anion radical scavenging activity of infested cucumber and chayote fruit tissue are depicted in Figure 1. The chayote INF tissue was 74.0% more active than APH 69.0% and HT 48.0%. The chayote healthy and apparent healthy samples showed high scavenging activity compared to infected tissue. Cucumber did not show any remarkable difference in activity. This indicates that chayote had more resistance and could remodel the damaged portion better than cucumber during the melon fly infestation.

**Figure 2:**
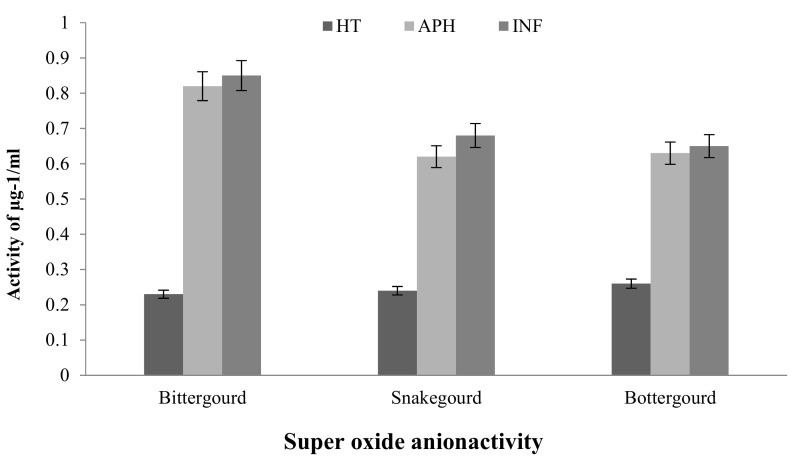
The superoxide anion antioxidant activity of melon fly infection n healthy (HT), apparently healthy (APH), and infected (INF) tissue extracts of cucurbit fruit. The vertical bars indicate the standard error. Significance at *p* ≤ 0.05.

The superoxide anion activity increased in bitter gourd, snake gourd, and bottle gourd infected tissue compared to healthy tissues. The increase was almost 150–200%, which is very significant and indicates the possible efficient mechanism of free radical scavenging during infection compared to healthy tissue (Figure 2).

#### 2.2.2. DPPH Free Radical Scavenging Assay

**Figure 3:**
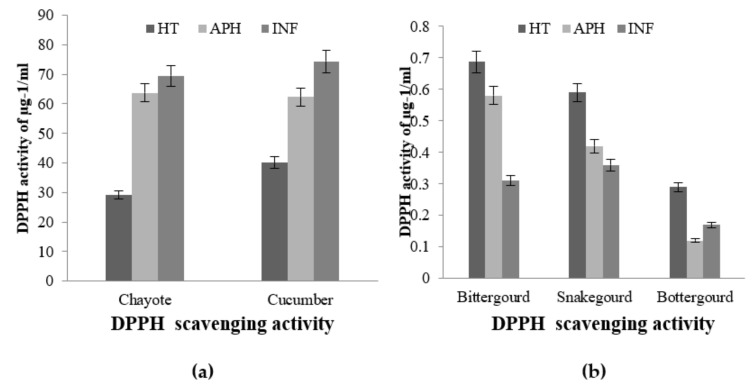
DPPH scavenger of a free radical assay from healthy (HT), apparently healthy (APH), and infected (INF) tissues: (**a**) cucumber and chayote fruit tissues; (**b**) bitter gourd, snake gourd, and bottle gourd during melon fly infestation. The vertical bars indicate the standard error. Significance at *p* ≤ 0.05.

A DPPH antioxidant activity was performed for infected and healthy tissues of the cucurbit fruits. The results are shown in Figure 3. DPPH scavenger activity for cucumber fruit tissue increased significantly in infected tissue, and the radical scavenging activity of infected tissue was observed to be 42.5% compared to healthy tissues (30%). Chayote tissue showed a remarkable increase of 65% during infection and was moderate while apparently healthy (60%) and relatively low during healthy (44%) conditions. The healthy bitter gourd and snake gourd tissues had more antioxidant potential compared to infected tissue parts. The antioxidant potential exhibited by tissue phytoconstituents decreased by almost 20–25% in apparently healthy and infected tissues. However, the bottle gourd infected tissue showed a marginal increase in antioxidant activity measured by DPPH assay (Figure 3).

### 2.3. Enzymatic Antioxidant Potential through Expression of PPO, SOD, and POX in Infected and Healthy Cucurbit Fruits on Melon Fly Infestation

In the healthy (HT), apparent healthy (AH), and infected (INF) tissue extracts of bottle gourd, snake gourd, and bitter gourd, changes in the activities of polyphenol oxidase, peroxidase, and superoxide dismutase were investigated. After *B. cucurbitae* infection, bottle gourd revealed high levels of antioxidant enzyme expression in infected tissues when compared to infected snake and bitter gourd tissues.

**Figure 4:**
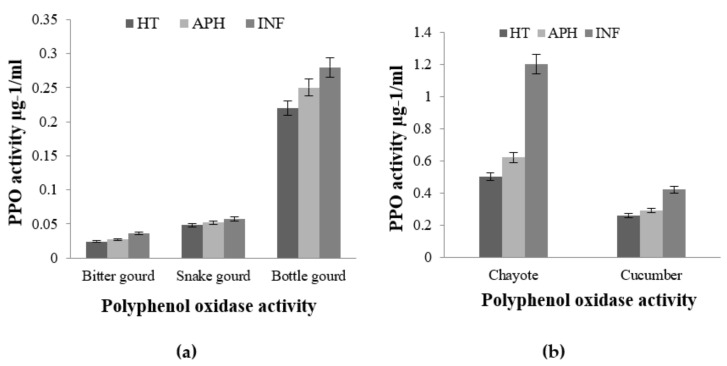
Polyphenol oxidase (PPO) activities of selected healthy (HT), apparently healthy (APH), and infected (INF) cucurbit fruit of Bitter gourd, Snake gourd and Bottle gourd (**a**) and Chayote and Cucumber (**b**) upon melon fly infestation. The vertical bars indicate the standard error. Significance at *p* ≤ 0.05.

The PPO enzyme activity was very low in bitter gourd and snake gourd, and there was no significant difference between healthy and infected tissues (Figure 4). This indicates that snake gourd and bitter gourd did not have high polyphenols, which are potential substrates for polyphenol oxidase and also have good antioxidant potential. However, in contrast, the bottle gourds had remarkably high PPO activity in both healthy and infected tissues (Figure 4). This infers the presence of high polyphenol content in bottle gourd tissues, and it had high antioxidant potential. This shows that the bottle gourd protects the tissue formation of polyphenol adducts upon activation of PPO expression, provides resistance against infecting larvae, and prevents tissue damage.

**Figure 5:**
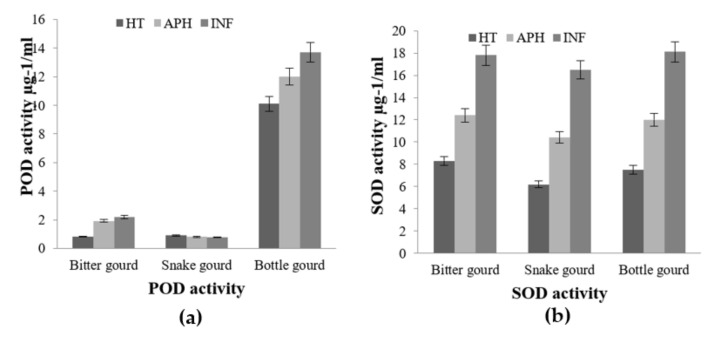
Comparison of peroxide (POD) and superoxide dismutase (SOD) enzyme activity in healthy (HT), apparently healthy (APH), and infected (INF) tissues of selected cucurbit fruit Bitter gourd, Snake gourd and Bottle gourd (**a**) and Chayote and Cucumber (**b**) upon melon fly infection. The vertical bars indicate the standard error. Significance at *p* ≤ 0.05.

When compared to apparently healthy and healthy tissues, bitter gourd, snake gourd, and bottle gourd infected tissues had a high level of superoxide dismutase (SOD) activity. However, there was no significant variation in SOD activity between healthy and infected bitter, snake, and bottle gourd tissues. All three of the cucurbit fruits showed an almost equal increase in enzyme activity during melon fly infestation (Figure 5).

**Figure 6:**
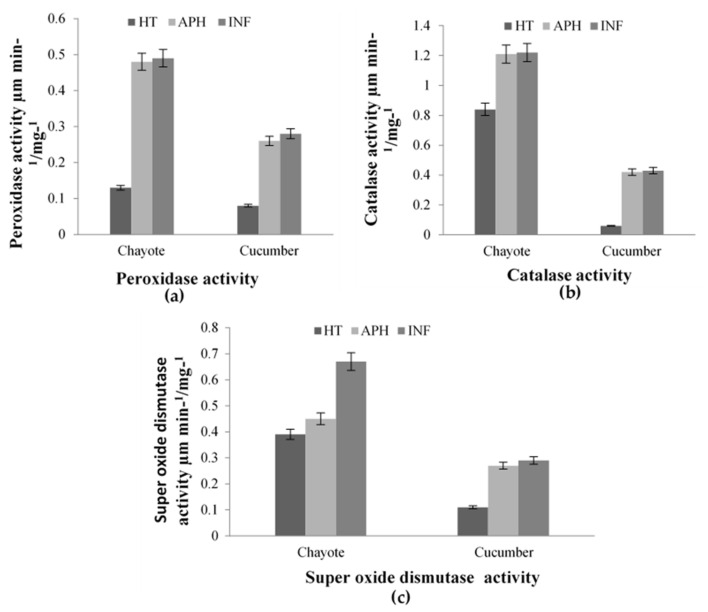
(**a**) Peroxidase (POX), (**b**) catalase (CAT), and (**c**) superoxide dismutase (SOD) antioxidant enzyme activity of melon fly infection in healthy (HT), apparently healthy (APH), and infected (INF) tissues of cucurbit and chayote fruit. The vertical bars indicate the standard error *p* = 0.05.

In the case of peroxide activity, this observation is in contrast to SOD activity, POD activity was found to be remarkably high in bottle gourd compared to bitter and snake gourds. There was a marginal increase in the POD activity of infected bottle gourd tissue compared to its healthy counterpart. However, there was no significant difference in the POD activity of bitter gourd’s and snake gourds healthy and infected tissues. These results indicate that there may be more of ROS scavenging activity in these tissues compared to peroxide production. This also gives clear evidence that the bottle gourd tissue samples had more antioxidant potential compared to the bitter gourd and snake gourd samples and suggests the possibility of more resistance and remodeling power in bottle gourd compared to the other two fruits during melon fly infestation (Figure 6).

Increased POD activity was noticed in chayote infected tissue than apparent healthy and healthy tissues. There was a moderate rise in the activity of cucumber infected tissue as that of healthy tissue. Similarly, infected chayote tissue exhibited excellent CAT activity compared to its healthy counterpart and cucumber tissues (Figure 6). The rise in activity of both POX and CAT was low in cucumber infected tissue compared to infected chayote tissue. The SOD showed an increased activity of 39.16% in infected chayote tissue compared to apparently healthy and healthy tissues (Figure 6).

### 2.4. Assay of the Non-Enzymatic Antioxidant Potential of Cucurbit Fruit Extracts

#### Ferric-Reducing Antioxidant Power (FRAP) and Superoxide Anion (o2–) Assay

**Figure 7:**
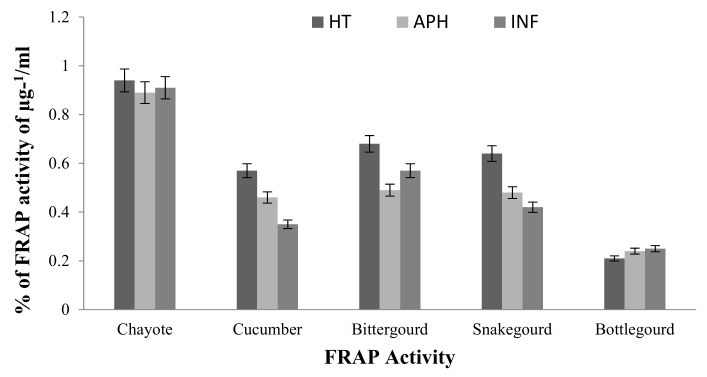
FRAP and superoxide anion antioxidant activity of healthy (HT), apparently healthy (APH), and infected (INF) cucurbit fruit tissues (bitter gourd, snake gourd, and bottle gourd) upon melon fly infestation. The vertical bars indicate the standard error. Significance at the *p* ≤ 0.05.

A FRAP antioxidant activity was conducted for infected and healthy tissues of the cucurbit fruits, and the results are shown in Figure 7. The healthy bitter gourd and snake gourd tissues had more antioxidant potential compared to infected tissue parts. The antioxidant potential exhibited by tissue phyto-constituents decreased by almost 20–25% in apparently healthy and infected tissues. However, the bottle gourd infected tissue showed a marginal increase in antioxidant activity measured by FRAP assay.

The results indicate the selective and differences in antioxidant molecules that are expressed during melon fly infestation in these cucurbit fruit tissues. It also reports the possibility of inducing high oxidative stress in the infected tissues, which imbalances the ratio of free radicals and antioxidants during infection in the damaged tissues.

In comparison to the other two cucurbits, the enzyme levels in apparently healthy bottle gourd tissues were significantly higher. The decreased antioxidant enzyme levels in snake gourd and bitter gourd made them more susceptible to melon fly infestation. As measured by DPPH, hydrogen peroxide, and superoxide scavenging assays, the infected and apparently healthy tissue of snake gourd and bitter gourd had weak ROS scavenging potential, whereas the infected and apparently healthy tissues of bottle gourd had strong ROS detoxification potential. Thus, the present study clearly showed decreased antioxidant enzymes and ROS activity in the vulnerable snake gourd and bitter gourd while increased levels in the bottle gourd.

## 3. Discussion

Melon fruit fly displayed nearly normal growth and development at 24 °C. Increased antioxidant and secondary metabolite levels had no effect on the growth and development of melon fruit flies. However, the amount of phenols, tannins, sugars, and proteins in the selected cucurbit vegetables and their varieties substantially impacted resistance or susceptibility to infestation. Phenols and tannins help cucurbit fruits defend against melon fruit fly attack [18], whereas sugars and proteins make cucurbits prone to fruit fly damage [19]. Estimates of antioxidant enzyme activity, such as POD, SOD, PPO, and CAT, found that resistant fruits have higher enzyme activity than susceptible fruits.

The responsiveness of cucurbit fruits to *B. cucurbitae* infection was investigated in this study by examining the activity of defensive enzymes and antioxidant activity in infected and healthy tissues. Chayote and bottle gourd have acquired a significant resistance to *B. cucurbitae* infestation by inducing and accumulating PPP enzymes upon infection [20]. Increased generation of ROS and subsequent accumulation of ROS scavenging enzymes and metabolites reflected the protective and signaling mechanisms in the infected fruits [21]. The strong correlation between phenolic acid content and antioxidant capacity in vegetables suggests that they play a key role in the bioactive properties of those plant products [22]. Antioxidative mechanisms protect fruit tissues from the lipid peroxidation at infection sites by preventing the spreading of necrotic lesions [23]. The levels of SOD, catalase, and antioxidant potential in melon fly infected tissues were measured in order to better understand the role of fruit’s protective antioxidative systems in response to pathogen attacks, particularly during infection, and to also assess the increased initiation of oxidative mechanisms.

This study discovered that *B. cucurbitae* infected and apparently healthy snake gourd and bitter gourd tissues had much higher SOD, peroxidase, and catalase levels than normal healthy tissue. Increased activity of catalase and SOD could be considered a sign of stress-induced H_2_O_2_ and O_2_ production [24]. Increased total antioxidant activity, superoxide scavenging activity, and DPPH assay indicated higher scavenging activity in both infected and apparently healthy tissues. Though antioxidant enzyme activity was upregulated in response to infection, it was lower than bottle gourd antioxidant activity, which could explain the bottle gourd’s high level of resistance to melon fly infestation [25]; however, experimental evidence has shown that ROS are generally resistant against infestation but not necessarily against pathogens [23].

Melon fly infestation of resistant fruits induces increased production of O_2_ radicals. Those boost a remarkable increase in the expression of anti-oxidative enzymes and molecules in infected tissues, which balances and neutralize the oxidative impact of melon, fly infestation as seen in chayote and bottle gourd [26]. In comparison to resistant fruit, the number of antioxidants produced in response to levels of O_2_ radicals produced in susceptible fruit was significantly lower. Excessive amounts of these free radicals have been responsible for the destruction of plant cells through the peroxidation of lipids and the production of secondary cytotoxic species in response to melon fly infestation [27]. High expression of PPO converts free polyphenols into polymeric, which are toxic to melon fly larvae in infected tissue and help provide resistance and defend against infecting larvae [25]. PPO activity was observed more in resistant fruits but not in susceptible varieties, hinting at the difference in the biochemical and physiological characteristics of resistance and susceptible cucurbits fruits.

Superoxide dismutase is an enzyme that neutralizes the superoxide anion, and it can cause significant cellular and molecular damage. In infested tissue, increased SOD activity is considered a vital factor in the antioxidant defensive system. It controls the amount of superoxide radicals and H_2_O_2_ generation, both of which have been found to act directly or indirectly in the plant defense system’s signal transduction [28,29].

## 4. Hypothetical Schematic Representation of the Antioxidant-Dependent Mechanism of Cucurbit Fruits against Melon Fly Infestation

**Figure 8:**
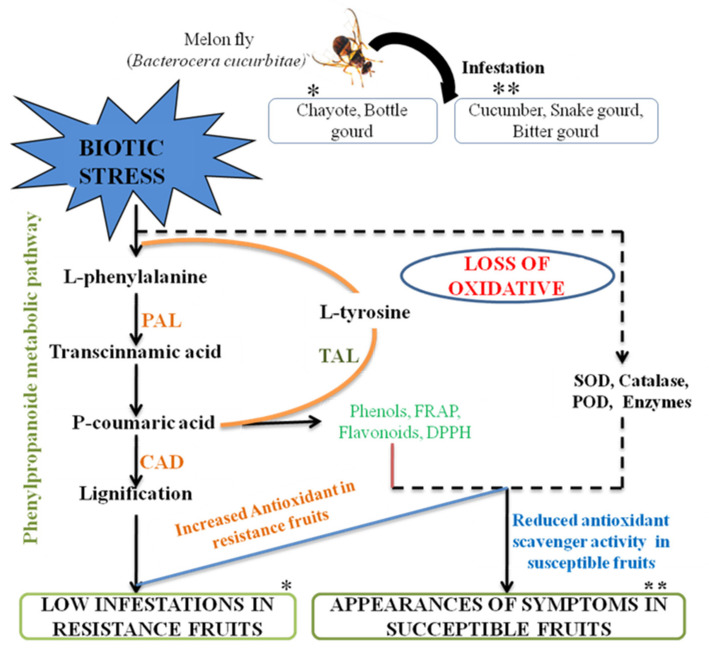
The phenylpropanoid pathway is a hypothetical model for the upregulation and ROS enzymes in response to biotic stress induced by melon fly infection. * Resistance; ** Susceptible.

In general, infestation by external pathogens induces biotic stress at the site of damage, which elicits the expression of many enzymes, such as SOD, CAT and POX, that efficiently scavenge and neutralize the oxidative damage to the cell during infection by converting the free aromatic amino acids into functional intermediates including phenolic and flavonoid compounds of plant origin [25]. The phenols and flavonoids such produced will have high antioxidant potential and bring about non-enzymatic antioxidant activity [30]. Infestations of melon fly by biotic stress activate the PPP, leading to the synthesis of trans-cinnamic acid from l-phenylalanine. The trans-cinnamic acid produced synthesizes coumaric acid. P-coumaric acid in the presence of CAD enzymes will lead to lignification in resistant fruits. Due to the oxidative burst (H_2_O_2_, O_2_) after melon fly infestation, there will be a low amount of lignification syntheses in the susceptible fruits. With this literature and based on our present studies, the below hypothesis represents the possible antioxidant mechanism imparted by cucurbit fruits in response to melon fly infestation (Figure 8).

## 5. Materials and Methods

Seeds of chayote, cucumber, bitter gourd, bottle gourd, and snake gourd were collected from the Department of Horticulture, University of Agricultural and Horticultural Sciences, Shivamogga, Karnataka, India. The seeds were sown in pots, and the fruits were harvested at maturity. Later, the fruits were artificially infected by oviposition of melon fly eggs collected from the Division of Entomology, Indian Institute of Horticulture Research (IIHR), Bangalore, India. The majority of the chemicals used in these experiments were from Hi-Media and Merck. Analytical grade chemicals and solvents were used throughout the experiment.

### 5.1. Assessment of Melons Fly Infestation on Cucurbit Fruits

The melon fly infestation was tested on freshly harvested matured cucurbit fruits by artificial oviposition. On the surface of each fruit, a circular hole measuring 5 mm in diameter and 2 mm in depth was punched using a steel punch and oviposition with 8–10 melon fly eggs. Melon fly larvae were developed by allowing the eggs to hatch [31]. The larva was allowed to feed on fruit to increase its size and turn into a pupa. As described by Shivashankar et al. [32], tissue sampling was employed to assess the extent of infestation and tissue damage by melon fly. Further, the melon fly infested cucurbits were grouped in to three categories viz., infected (melon fly infestation tissue), apparently healthy (moderated infestation tissue), and healthy (without infestation tissue) based on tissue damage and larval population on the infected area.

### 5.2. Estimation of Total Flavonoid and Phenolic Contents

The total phenolic content was determined using the procedure proposed by Alafiatayo et al. [33]. Using gallic acid as a standard, the total phenolic content was calculated. Breifly, 100 µL of tissue samples were mixed with 8.9 mL of distilled water and 1 mL of Folin–Ciocalteu’s (FC) reagent. After 5 min of shaking, 10 mL of 7% Na_2_CO_3_ and 4.9 mL of distilled water were added for a total volume of 25 mL. The absorbance was measured at 750 nm after 90 min of incubation at room temperature. Total phenolic acid concentrations in infected and healthy samples were later reported as g/mL of gallic acid equivalents (GAEs).

The total flavonoid concentration was determined using a quercetin standard in the aluminum chloride colorimetric assay [34]. An aliquot (1 mL) of tissue extract was added to a 10 mL volumetric flask containing 4 mL distilled water. After five min, 0.3 mL 5 percent NaNO_2_ was added to the flask, followed by 0.3 mL 10 percent AlCl_3_. After five min, 2 mL 1M NaOH was added, followed by distilled water to increase the volume to 10 mL. The absorbance was measured at 510 nm against a blank after the solution was mixed. In milligs per milliliter of quercetin equivalents, the total flavonoid content was determined (QE).

### 5.3. Assay of Non-Enzymatic Antioxidant Potential of Cucurbit Fruit Extracts 

#### 5.3.1. FRAP Activity

A 5 g healthy and infected fruit sample was homogenized and filtered through muslin cloth with l M, 100 mL sodium acetate buffer (pH 3.6). One hundred liters of filtered sample were mixed with 3 mL FRAP reagent (containing 2,4,6-tripyridyl-s-triazine (TPTZ), l M HCl, and l M FeCl_3_ in a 10:1:1 ratio) and tubes were vortex and incubated in a boiling water bath for 30 min at room temperature, after which absorbance was measured at 593 nm with a UV-visible spectrophotometer (Perkin Elmer, UV-Vis, LAMBDA 365, Waltham, MA, USA) [7]. The results were expressed in terms of µM Fe^2+^ equivalents [35].

#### 5.3.2. Superoxide Anion (o2–) Activity

Superoxide anion activity from different cucurbit infested fruit tissue was homogenized with PBS pH 7.2 and superoxide scavenging activity is followed by Doke [36]. One hundred milliliters of tissue homogenate were suspended in a solution containing 3 mL Tris-HCl buffer (16 mM, pH 8.0), 1 mL NBT (50 mM), 1 mL NADH (78 mM), and 1 mL PMS (10 mM). At 30 °C for 10 min, the mixture was incubated, and the absorbance was measured at 560 nm. Ascorbic acid was utilized as a standard.

The following equation was used to determine scavenging ability:

$$Scavenging\;effect\;\%\;=\frac{¼\;\left(1-Absorbance\;of\;sample\right)}{\left(Absorbanceofcontrol\right)}\times \;100\;$$

#### 5.3.3. DPPH Activity

The scavenging activity of the fractions was measured by 2,2-diphenyl-1-picrylhydrazyl (DPPH) assay as per the procedure outlined by Anwesha et al. [37]. In brief, various sample concentrations of 20, 40, 60, 80, and 100 µL were mixed with 1.5 mL of DPPH. The mixture was agitated and set aside for 30 min at room temperature in the dark. Later, absorbance was measured at 517 nm. Similarly, control was prepared without a sample solution. The scavenging activity was assessed based on the percentage of DPPH radicals scavenged, and the scavenging impact (%) was computed using the following equation.

$$Scavenging\;effect\;\%\;=\frac{\left(1-Absorbanceofsample\right)}{\left(Absorbance\;of\;control\right)}\times \;100$$

### 5.4. Activity of Defensive Enzymes Activity of Cucurbit Fruit Extracts

Cucurbit fruit tissue samples were taken from healthy and infected portions. Ten g of tissue samples were extracted by homogenation with 100 mM phosphate buffer (pH 7.2). At 10 °C, the homogenate was centrifuged for 15 min at 10,000 rpm. Later, with the collected supernatant, antioxidant enzyme activity was determined.

#### 5.4.1. Polyphenol Oxidase (PPO) Assay

The PPO activity was estimated as per the procedure described by Liu et al [38]. In a 10 mL test tube, 1.5 mL of 40 mM catechol and 2.3 mL of 0.1 M phosphate buffer (pH 6.5) were combined in a standard reaction mixture and incubated for 5 min at 25 °C in a water bath. The test tube was then filled with 0.2 mL of crude enzyme and properly mixed. Later immediately, a UV spectrophotometer was used to measure the change in absorbance at 420 nm and a further linear section of the curve was used to calculate PPO enzyme activity. One unit of PPO activity was defined as the amount of enzyme that caused an increase in absorbance of 0.001 per min.

#### 5.4.2. Peroxidase (POD) Assay

Approximately 100 g of cucurbit fruit tissue was mixed with 10 mL of phosphate buffer (pH 7.0) and well homogenized with a pestle and mortar. The extract was centrifuged for 15 min at 10,000 rpm. POD activity was performed according to [39]. One and a half milliliters of 67 mM phosphate buffer pH 6.0 and 0.5 mL of the sample were pipetted out into a 10 mM path length polyacrylic cuvette and incubated for 1 min at 25 °C. Later, 0.2 mL of a 1.7 mM ABTS solution and 0.2 mL of a 0.8 mM hydrogen peroxide solution was added and stirred to begin the reaction. Absorbance was measured at 405 nm using a spectrophotometer. Further, the quantity of enzyme that resulted in an increase of 0.01 per min in absorbance was measured as one unit of peroxidase.

#### 5.4.3. Superoxide Dismutase Activity (SOD)

The ability of SOD to prevent the photochemical degradation of NBT was measured using the method described in [40]. Fifty microliter samples were added to a 3 mL reaction mixture that contained 50 mM phosphate buffer (pH 7.8), 13 mM methionine, 75 M NBT, 2 M riboflavin, and 0.1 mM EDTA. At the end, riboflavin was added to the tubes and mixed well. A 15 W fluorescent bulb was used to illuminate the solution in a 10 mL beaker for 10 min in an aluminum foil enclosed box [41]. Later, the absorbance of the reaction mixture was measured at 560 nm. SOD activity was measured as the quantity of enzyme required to prevent NBT from being reduced by 50%, and it was measured in units per mg of protein.

#### 5.4.4. Catalase Assay (CAT)

Cucurbit fruit tissues were collected and homogenized using a buffer (sodium phosphate, 0.1 M, pH 7.2) and PVPP (polyvinyl pyrophosphate) concentration of 100 g fresh tissue weight. Catalase activity was determined as given in [25]: 20–100 µL of enzyme sample processed from different cucurbit fruits tissue were taken, and 8 mL 67 mM phosphate buffer (PH 7.0) and 50 µL of 240 mM of hydrogen peroxide were added. The absorbance at 240 nm after vortexing the mixture was measured. The catalase activity was measured in units per mg of proteins and expressed as the quantity of enzymes that decomposed 1 μM H_2_O_2_ per min at 25 °C.

### 5.5. Statistical Analysis

The data were analyzed using SPSS V 25.0 statistical software. A simple paired *t*-test was employed to compare the mean values of antioxidants and defensive enzymes between the resistant and susceptible species. The least significant difference (LSD) was employed to compare treatment means at the 5% (*p* < 0.05) and 1% (*p* < 0.01) level of significance. To assess the difference between treatment means in the case of significant results, the critical difference was determined at the 5% and 1% level of probability.

## 6. Conclusions

Melon fly is a serious pest of cucurbits vegetables, as it directly and indirectly causes damage to cucurbit fruits in the early stage of its development. The infection of fruit tissues induces increased oxidative damage to the cell, which is mediated by producing reactive oxygen species. The role of an antioxidant in processes that involve protecting (resistant) fruits by biotic and abiotic stresses induced by ROS has been the subject of extensive studies. In our study, melon fly infestations in resistant fruits expressed high antioxidant and enzyme activity levels that were closely associated with scavenging ROS. The bottle gourd and chayote had a high magnitude of antioxidant defensive mechanism compared to the other three susceptible fruits during melon fly infestation. On the contrary, in bitter gourd, snake gourd, and cucumber tissues, the lignin and total phenol contents and antioxidant defensive mechanism were very trivial for which reason they could not exhibit any defense to melon fly infestation and were inclined to sustain damage. The levels of antioxidant and enzyme activity expressed due to the melon fly infestation were identified as the factors that strengthen the confrontation of the synthesis of specific bioactive molecules that may induce resistance in cucurbit fruits.

## Figures and Tables

**Table 1:** Total phenolic and flavonoid contents and DPPH activity in healthy, apparently healthy, and infected tissues during melon fly infection to combat infection-induced biotic stress (mean ± SD).

Parameter	Chayote			Cucumber			Bitter Gourd			Snake Gourd			Bottle Gourd		
	HT	APH	INF	HT	APH	INF	HT	APH	INF	HT	APH	INF	HT	APH	INF
TPC GAE (µg/mL)	0.731 ± 0.01	0.683 ± 0.01	0.657 ± 0.01	0.462 ± 0.05	0.418 ± 0.05	0.408 ± 0.05	0.537 ± 0.01	0.483 ± 0.01	0.371 ± 0.01	0.485 ± 0.05	0.425 ± 0.05	0.340 ± 0.02	0.824 ± 0.02	0.715 ± 0.05	0.695 ± 0.01
TFC GAE (µg/mL)	0.937 ± 0.01	0.913 ± 0.01	0.894 ± 0.01	0.375 ± 0.11	0.328 ± 1.01	0.276 ± 0.01	0.408 ± 1.01	0.353 ± 0.01	0.327 ± 0.01	0.27 ± 0.11	0.248 ± 1.06	0.196 ± 0.03	0.686 ± 0.01	0.652 ± 0.01	0.637 ± 0.01
IC50 DPPH	81.35 ± 0.01	78.61 ± 0.01	78.14 ± 0.01	62.42 ± 0.02	58.17 ± 0.01	55.86 ± 0.02	63.24 ± 0.03	59.37 ± 0.05	56.51 ± 0.01	49.75 ± 0.03	42.67 ± 0.02	41.52 ± 0.02	73.86 ± 0.05	72.46 ± 0.05	71.52 ± 0.05

## Data Availability

Data are available upon request from the first author.

## Abbreviations

HT: healthy tissue; APH: apparently healthy, INF: (infested) tissue; DPPH: 2,2-diphenyl-1-picrylhydrazyl; FRAP: ferric-reducing antioxidant power; PPO: polyphenol oxidase; POD: peroxidase; ROS: reactive oxygen species; SOD: superoxide dismutase; CAT: catalase; FC: Folin–Ciocalteu; QE: quercetin equivalent; TPTZ: 2,4,6-tripyridyl-s-triazine; PMS: phenazine metho sulphate.
